# Unlocking the Neuroprotective Effect of Quercetin Against Cadmium-Induced Hippocampal Damage in Rats: PPARγ Activation as a Key Mechanism

**DOI:** 10.3390/ph18050657

**Published:** 2025-04-29

**Authors:** Doha M. Al-Nouri

**Affiliations:** Department of Food and Nutrition Sciences, College of Food and Agriculture Sciences, King Saud University, Riyadh 11495, Saudi Arabia; dr_nouri@ksu.edu.sa

**Keywords:** quercetin, cadmium, hippocampus, memory, oxidative stress/inflammation, PPARγ

## Abstract

**Background:** This study investigates the effects of cadmium chloride (CdCl_2_) on hippocampal peroxisome proliferator-activated receptor gamma (PPARγ) expression and examines whether PPARγ activation mediates the neuroprotective effects of quercetin (QUR). **Methods:** Sixty adult male rats were included in this study, separated into 12 rats per group as follows: control, CdCl_2_ (0.5 mg/kg), CdCl_2_ + PPARγ agonist (Pioglitazone, 10 mg/kg), CdCl_2_ + QUR (25 mg/kg), and CdCl_2_ + QUR + PPARγ antagonist (GW9662, 1 mg/kg). Treatments were administered orally for 30 days. At the end of the experiment, behavioral memory tests, hippocampal histology, markers of cholinergic function, neuroplasticity, oxidative stress, inflammation, and apoptosis, as well as transcription levels of some genes were carried out. **Results:** CdCl_2_ exposure significantly reduced hippocampal PPARγ mRNA and DNA binding potential and nuclear levels. Additionally, CdCl_2_ impaired spatial, short-term, and recognition memory, decreased granular cell density in the dentate gyrus (DG), and reduced levels of neuroprotective factors, including Nrf2, brain-derived neurotrophic factor (BDNF), acetylcholine (ACh), and several antioxidant enzymes including heme-oxygenase-1 (HO-1) and superoxide dismutase (SOD), as well as reduced glutathione (GSH). Conversely, CdCl_2_ elevated levels of oxidative stress, inflammation, and apoptosis markers such as interleukin-6 (IL-6), malondialdehyde (MDA), Bax, tumor necrosis factor-α (TNF-α), and cleaved caspase-3. QUR and Pioglitazone reversed these effects, restoring expression and PPARγ activation, improving memory, and modulating antioxidant and anti-inflammatory pathways. In contrast, blocking PPARγ with GW9662 negated the neuroprotective effects of QUR, exacerbating oxidative stress and inflammation by reversing all their beneficial effects. **Conclusions:** Activation of PPARγ by QUR or Pioglitazone offers a promising therapeutic strategy for mitigating CdCl_2_-induced neurotoxicity.

## 1. Introduction

A wide range of neurological diseases involve the gradual degeneration and death of neurons within the central nervous system. Neurological diseases result in profound impairments in cognitive functions such as memory, learning, concentration, and overall intellectual ability [1]. Prominent examples include spinocerebellar ataxia, Huntington’s disease, dementia, Alzheimer’s disease (AD), Parkinson’s disease (PD), amyotrophic lateral sclerosis (ALS), prion disease, and spinal muscular atrophy [1,2,3]. The etiology of these disorders is multifactorial, with both genetic predispositions and environmental factors—such as diet, infections, pharmaceuticals, and environmental pollutants—playing significant roles in their onset and progression [2]. Over recent decades, cadmium (Cd) has emerged as one of the most prominent environmental pollutants implicated in neurodegeneration, particularly in AD, PD, and ALS [3,4,5,6,7,8].

Cd, a ubiquitous heavy metal, is readily absorbed into the body through various routes, including ingestion, dermal exposure, and inhalation, ultimately accumulating in the brain. This metal has increased exposure due to mining activities in the land, tobacco smoking, and its wide use in plastics, paints, and batteries [3]. Cd’s long biological half-life, averaging 23 years, coupled with its minimal urinary excretion, facilitates its accumulation and results in significant multi-organ toxicity [4]. In the central nervous system, Cd disrupts neuronal function by penetrating the blood–brain barrier (BBB), where it impairs neurogenesis, cholinergic function, synaptic plasticity, and neurotransmitter synthesis while simultaneously activating apoptosis pathways [3,5]. The precise mechanisms underlying Cd-induced neurotoxicity are still not fully elucidated but are thought to involve DNA damage, oxidative stress, neuroinflammation, and apoptotic signaling cascades [3,5,6].

Recent studies have highlighted the critical neuroprotective role of peroxisome proliferator-activated receptor gamma (PPARγ) in mitigating neurodegeneration [7,8]. The nuclear hormone receptor transcription factor, PPARγ, is distributed in the majority of the tissues and is highly expressed in the brain. This receptor regulates a wide range of genes involved in metabolic processes, immune responses, redox balance, inflammation, and apoptosis [7]. Importantly, dysregulation of PPARγ expression and activity has been observed in several neurological disorders, including AD, PD, stroke, ALS, and multiple sclerosis [7,8]. Activation of PPARγ has been shown to alleviate oxidative stress, modulate inflammation, prevent neuronal apoptosis, and enhance synaptic function, thereby offering a promising therapeutic strategy for various neurodegenerative conditions [7].

In recent years, plant-based compounds have gained considerable attention as potential treatments for neurological disorders due to their natural antioxidant and anti-inflammatory properties. Quercetin (QUR) is abundant in nuts commonly isolated from diverse vegetables and fruits and has demonstrated significant neuroprotective effects across a variety of preclinical models [9,10,11,12,13,14,15,16,17]. QUR has been shown to exert its protective effects through its potent antioxidant and anti-inflammatory actions, mediated via the regulation of key intracellular signaling pathways, including those involved in oxidative stress and inflammation [9,10]. Notably, QUR has been found to protect against Cd-induced hippocampal and cortical damage by scavenging free radicals, enhancing antioxidant capacity, activating Nrf2, inhibiting NF-κB, and improving synaptic transmission. A full review of the antioxidant and anti-inflammatory neuroprotective effects of QUR against CdCl_2_-induced toxicity is discussed in excellent studies and reviews [11,12,13,14,15,16,17].

While the molecular mechanisms underlying CdCl_2_-induced neurotoxicity have been well-established, the specific role of PPARγ in this process has never been systematically examined. This hypothesis is further supported by studies demonstrating that Cd exposure leads to the degradation of PPARγ in pulmonary macrophages [18], impairs PPARγ-mediated metabolic regulation in the liver [19], and induces nephrotoxicity by suppressing PPARγ activity in renal cells [20]. In the same manner, despite the growing body of evidence supporting QUR’s neuroprotective potential, the effects of CdCl_2_ on PPARγ expression and activation remain poorly understood, particularly in the context of neurodegeneration. Given QUR’s known antioxidant and anti-inflammatory properties, it is plausible that CdCl_2_ exposure may suppress PPARγ activity, thereby exacerbating oxidative stress and inflammation in the brain. We built this hypothesis based on the reported neuroprotective antioxidant and anti-inflammatory effects of QUR in the lung, liver, and heart of various diseased models due to PPARγ activation [21,22,23,24,25,26,27].

In this study, we aim to investigate the role of PPARγ in mediating CdCl_2_-induced memory dysfunction and hippocampal damage in rate and if the neuroprotective effects of QUR against such neurotoxicity are mediated by activating this transcription factor. The findings of this study demonstrate that Cd-induced neurotoxicity is associated with downregulation and reduced nuclear activity of PPARγ. Furthermore, we show that the full protective antioxidant and anti-inflammatory effect of QUR is mediated through the activation of PPARγ, highlighting its crucial role in regulating cholinergic function, synaptic activity, NF-κB, and the Nrf2/antioxidant axis. Importantly, these neuroprotective effects are completely abolished by GW9662, a selective PPARγ antagonist, providing compelling evidence of the pivotal role of PPARγ in modulating neuroinflammation and oxidative stress in Cd-induced neurotoxicity.

## 2. Results

### 2.1. Effects of QUR and Pioglitazone on Neurological Scores and Memory Function in Rats

**Figure 1:**
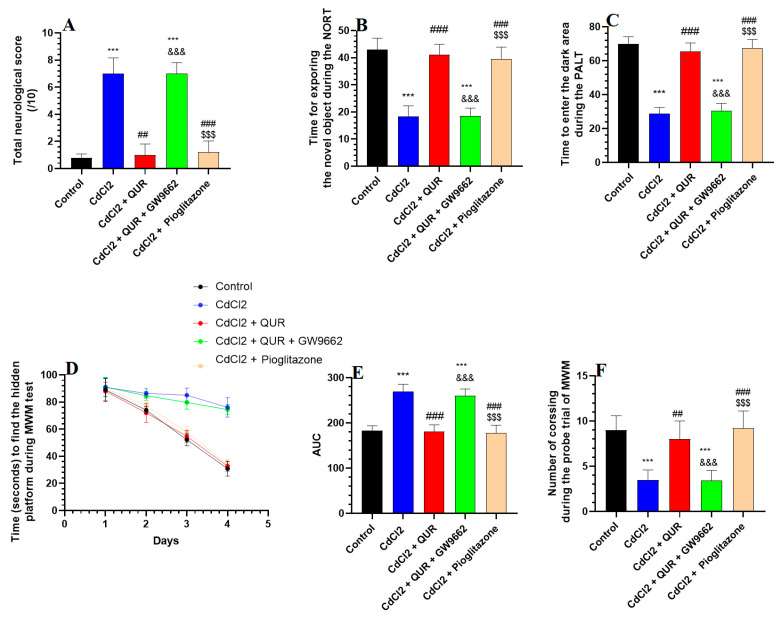
The total neurological score (**A**) alongside the outcomes of the novel object recognition test (NORT) (**B**), passive avoidance learning test (PALT) (**C**), and Morris Water Maze (MWM) (**D**–**F**) across all experimental rat groups. Data are expressed as mean ± SD (n = 8 per group). Statistical significance was determined at *p* < 0.05. ***: Compared to the control group (*p* < 0.001); ^##, ###^: Compared to CdCl_2_-treated rats (*p* < 0.001); ^&&&^: Compared to CdCl_2_ + QUR-treated rats (*p* < 0.001); and ^$$$^: Compared to CdCl_2_ + QUR + GW9662 (*p* < 0.001). QUR: Quercetin; GW9662: PPARγ inhibitor.

The total neurological score was significantly increased in CdCl_2_-treated rats compared to the control group (Figure 1A). CdCl_2_-intoxicated rats took less time to explore the novel object or enter the dark room during the NORT and PALT (Figure 1B and 1C, respectively). There were no significant variations in the time spent by the CdCl_2_-treated rats finding the hidden platform as measured on days 1, 2, 3, and 4 during the MWM test (Figure 1D). However, CdCl_2_-treated rats showed a significantly reduced area under the curve (AUC) for the corresponding swimming time taken to find the hidden platform on these days (Figure 1E). In addition, the CdCl_2_-intoxicated rats showed a significant reduction during the trial phase in the amount of time needed to cross over the platform (Figure 1F). The treatment with either QUR or Pioglitazone significantly lowered the total neuro-logical score, and these rats spent a longer time exploring the novel object or entering the dark area in the NORT and PALT (Figure 1A–C). They also exhibited a progressive significant decrease in the swimming times taken to locate the hidden platform during days 1–4, as well as a reduction in their AUC scores as compared to the CdCl_2_-treated rats (Figure 1D,E). The rats treated with CdCl_2_ + QUR and CdCl_2_ + Pioglitazone also undertook significantly more crossing trials to find the hidden platform (Figure 1F). No significant variations were shown between these parameters when the CdCl_2_ + QUR-treated rats were compared with the CdCl_2_ rats which were administered Pioglitazone. On the other hand, when the comparison was made against CdCl_2_ + QUR model rats, the neurological scores and the time needed to find the hidden platform were significantly increased, and the number of crossings over the hidden platform, the time needed to explore the novel subject, and the time taken to enter the dark area were significantly reduced in CdCl_2_ + QUR + GW9662 rats (Figure 1A–F). Notably, the mean values of all these measures were not significantly varied between CdCl_2_- and CdCl_2_ + QUR + GW9662 rats.

### 2.2. Effect on Hippocampal Levels of Markers of Inflammation and Oxidative Stress

As shown in Table 1, CdCl_2_ exposure led to significant elevation in MDA, 8-OHdG, TNF-α, IL-6, and RAGE levels, along with increased nuclear NF-κB in the hippocampus compared to control rats. Conversely, antioxidant markers such as GSH, SOD, and HO-1, as well as nuclear Nrf2, were markedly diminished in CdCl_2_-treated rats relative to the control group (Table 1). Co-treatment with either QUR or Pioglitazone effectively mitigated these effects, as demonstrated by lower MDA, 8-OHdG, TNF-α, IL-6, and RAGE levels, as well as reduced nuclear NF-κB, compared to the CdCl_2_-intoxicated group (Table 1). Simultaneously, hippocampal GSH, SOD, HO-1, and nuclear Nrf2 levels were significantly elevated in these treatment groups relative to CdCl_2_-exposed rats (Table 1). Interestingly, in QUR-treated rats, the levels of GSH, SOD, HO-1, and 8-OHdG, as well as nuclear Nrf2, were comparable to those observed in control animals (Table 1). However, introducing GW9662 alongside QUR reversed these protective effects, leading to a resurgence of MDA, 8-OHdG, TNF-α, IL-6, RAGE, and nuclear NF-κB levels, while simultaneously reducing GSH, SOD, HO-1, and nuclear Nrf2 when compared to CdCl_2_ + QUR- or CdCl_2_ + Pioglitazone-treated groups (Table 1). Notably, no significant differences were observed between the CdCl_2_-only group and the CdCl_2_ + QUR + GW9662-treated rats, indicating that GW9662 negated the beneficial impact of QUR (Table 1).

### 2.3. Effect on Hippocampal Levels of BDNF and Other Cholinergic Markers

As presented in Table 2, CdCl_2_ exposure led to a significant decline in hippocampal levels of ACh, BDNF, and ChAT, while AchE levels were markedly elevated compared to the control group. Treatment with either QUR or Pioglitazone effectively counteracted these alterations, resulting in significantly higher levels of ACh, BDNF, and ChAT, along with a notable reduction in AchE levels relative to CdCl_2_-exposed rats (Table 2). Notably, no significant differences were observed between the CdCl_2_ + QUR- and CdCl_2_ + Pioglitazone-treated groups. However, co-administration of GW9662 with QUR reversed these beneficial effects, as evidenced by a significant reduction in ACh, BDNF, and ChAT levels, along with a concomitant rise in AchE, compared to rats treated with CdCl_2_ + QUR alone (Table 2). Importantly, these values did not significantly differ from those observed in the CdCl_2_ model group, suggesting that GW9662 negated the protective effects of QUR (Table 2)

### 2.4. Effect on Apoptotic and Anti-Apoptotic Markers

**Figure 2:**
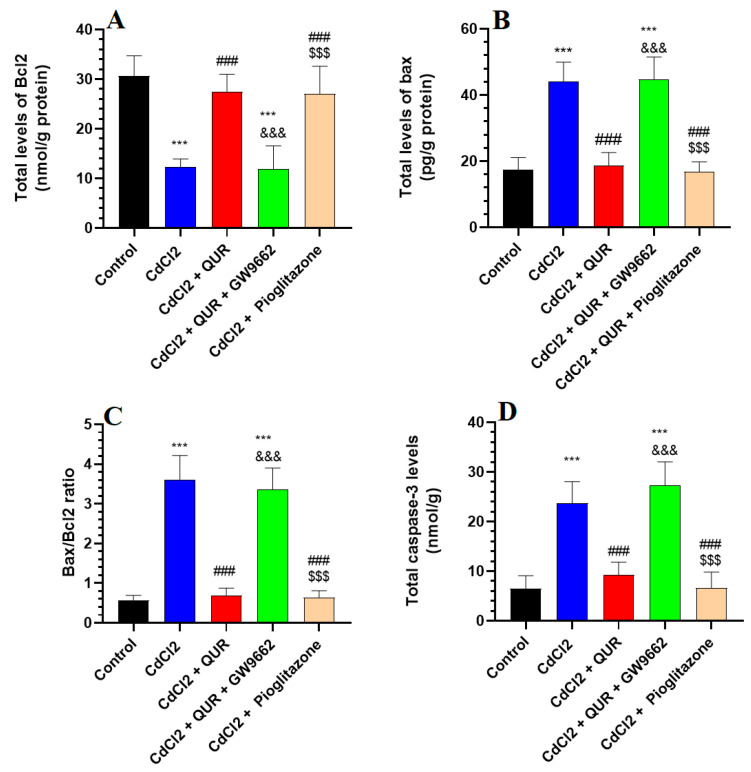
Hippocampal analysis of the levels of Bax, Bcl-2, Bax/Bcl-2 ratio (**A**–**C**), and caspase-3 (**D**) across experimental groups. The values were significantly different at *p* < 0.05. ***: Compared to control (*p* < 0.05, 0.01, & 0.001 Statistical significance was determined at *p* < 0.05. ***: Compared to the control group (*p* < 0.001); ^###^: Compared to CdCl_2_-treated rats (*p* < 0.001); ^&&&^: Compared to CdCl_2_ + QUR-treated rats (*p* < 0.001); and ^$$$^: Compared to CdCl_2_ + QUR + GW9662 (*p* < 0.001).

As illustrated in Figure 2A–D, CdCl_2_ exposure resulted in a significant decrease in hippocampal Bcl-2 levels, accompanied by an increase in caspase-3 and Bax expression, as well as an elevated Bax/Bcl-2 ratio, when compared to the control group. However, treatment with either QUR or Pioglitazone effectively counteracted these changes, leading to significantly higher Bcl-2 levels and lower caspase-3 and Bax levels, along with a reduced Bax/Bcl-2 ratio relative to CdCl_2_-exposed rats (Figure 2A–D). No statistically significant differences were observed among these treatment groups or in comparison to control rats. In contrast, co-administration of GW9662 with QUR led to a notable decline in Bcl-2 levels, paralleled by reductions in Bax, caspase-3, and the Bax/Bcl-2 ratio, compared to CdCl_2_ + QUR-treated rats (Figure 2A–D). Importantly, these apoptotic markers in the CdCl_2_ + QUR + GW9662 group were not significantly different from those observed in CdCl_2_-treated rats, indicating that GW9662 abolished the protective effects of QUR (Figure 2A–D).

### 2.5. Effect on the mRNA and Nuclear Activity of PPARα

**Figure 3:**
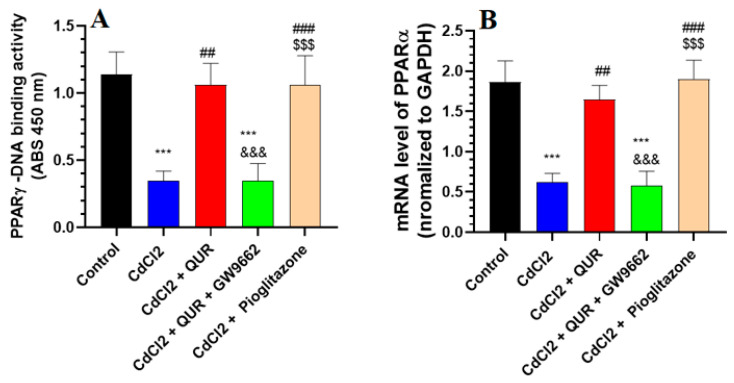
Nuclear DNA binding activity (**A**) and mRNA levels (**B**) of PPARα in the hippocampi of all groups of rats. The values were significantly different at *p* < 0.05. ***: Compared to control (*p* < 0.05, 0.01, & 0.001 Statistical significance was determined at *p* < 0.05. ***: Compared to the control group (*p* < 0.001); ^##, ###^: Compared to CdCl_2_-treated rats (*p* < 0.001); ^&&&^: Compared to CdCl_2_ + QUR-treated rats (*p* < 0.001); and ^$$$^: Compared to CdCl_2_ + QUR + GW9662 (*p* < 0.001).

As depicted in Figure 3A,B, CdCl_2_ exposure led to a pronounced decline in both the mRNA expression and nuclear DNA binding activity of PPARα in the hippocampus compared to control rats. Conversely, a substantial upregulation in PPARγ transcript levels and its DNA binding activity was evident in the hippocampi of rats receiving either CdCl_2_ + QUR or CdCl_2_ + Pioglitazone treatment (Figure 3A,B). Notably, no significant discrepancies in PPARγ expression or nuclear binding activity were detected between these two treatment groups or in comparison to control animals. However, the introduction of GW9662 alongside QUR reversed these molecular alterations, resulting in a marked suppression of PPARγ mRNA expression and nuclear DNA binding activity relative to the CdCl_2_ + QUR-treated group (Figure 3A,B). Importantly, PPARγ transcript levels and nuclear activity in the CdCl_2_ + QUR + GW9662-treated rats were statistically indistinguishable from those observed in the CdCl_2_-only group, indicating a complete abrogation of QUR’s modulatory effects by GW9662 (Figure 3A,B).

### 2.6. Effect on Dental Gyrus Histopathology

**Figure 4:**
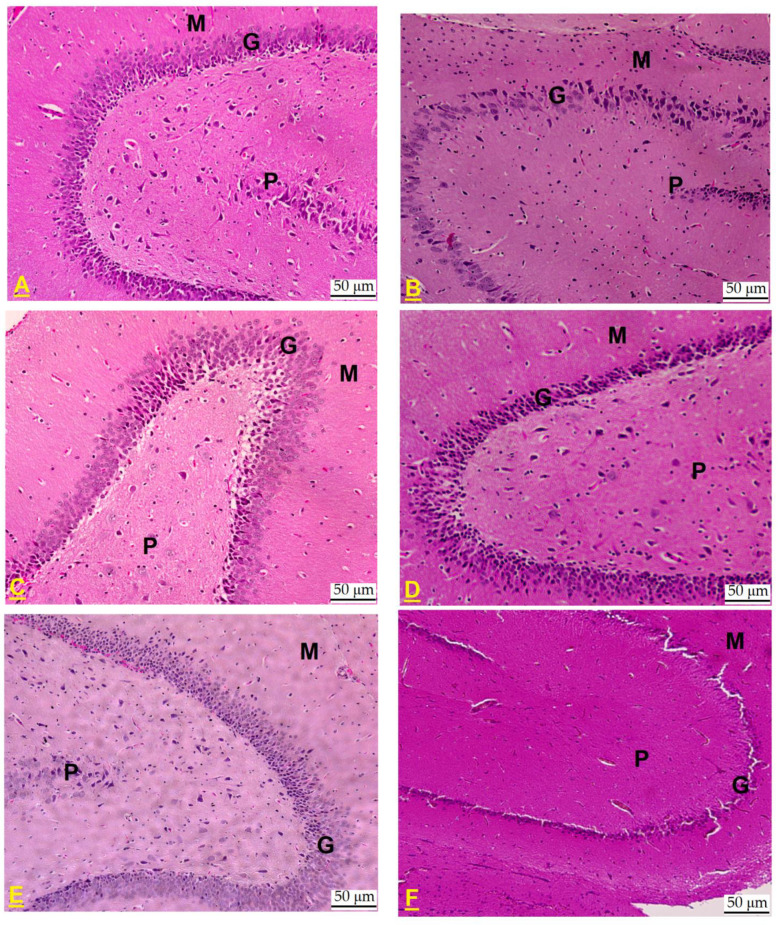
Histological alterations in the dentate gyrus (DG) of the hippocampus across experimental rat groups. (**A**) The DG from a control rat displayed normal architecture, with distinct polymorphic (P), granular (G), and molecular (M) layers. The granular layer consisted of intact, round cells organized in five to eight layers. (**B**) In contrast, the DG from a CdCl_2_-treated rat exhibited a notable reduction in the number of cells within the granular layer, now comprising only two to three layers. A majority of the cells displayed pyknosis, indicative of cellular degeneration. (**C**,**D**): taken from a CdCl_2_ + QUR-treated rat, which exhibited a normal DG with six to eight granular layers of healthy intact cells. (**E**) Taken from a CdCl_2_ + Pioglitazone-treated rat, it showed an almost normal structure with a normal number of cells forming the granular layer. (**F**) Taken from a CdCl_2_ + QUR + GW9662-treated rat, showing similar damage in the granular layer with an increased number of pyknotic cells. Hematoxylin and Eosin, 200×.

In the hippocampal dentate gyrus (DG) of untreated control rats, the structural integrity remained intact, exhibiting its characteristic tri-layered organization comprising the polymorphic, granular, and molecular layers. The granular layer comprises intact round cells of five to eight layers (Figure 4A). In the DG of CdCl_2_-exposed rats, a marked decline in the cellular density of the granular layer was observed, with the layer thinning to only two to three cell layers. Additionally, a majority of the cells exhibited pyknosis, indicative of nuclear condensation and cellular degeneration (Figure 4B). On the other hand, almost normal DGs with six to eight granular layers of healthy intact cells were observed in the hippocampi of CdCl_2_ + QUR- (Figure 4C,D) and CdCl_2_ + Pioglitazone-treated rats (Figure 4E). Similarly, the DG of the hippocampi from CdCl_2_ + QUR + GW9662-treated rats exhibited comparable damage, characterized by an increased presence of pyknotic cells, further exacerbating the deterioration of the granular layer (Figure 4F).

## 3. Discussion

**Figure 5:**
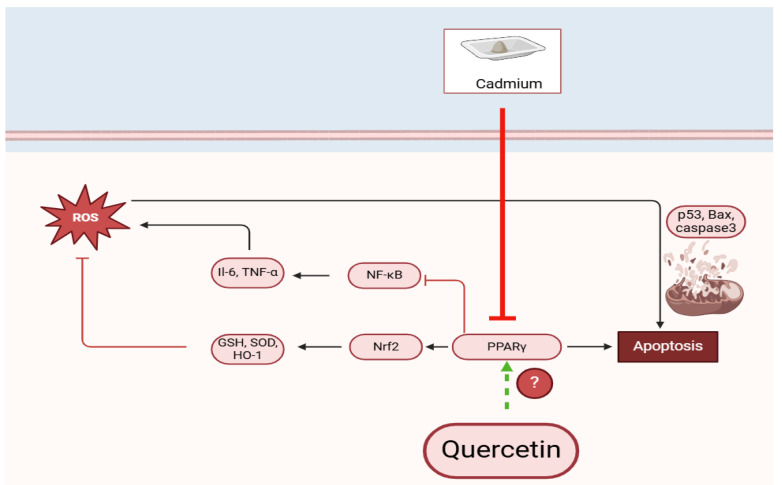
A graphic abstract demonstrates the precise neuroprotective effect of quercetin against cadmium-induced hippocampal damage in rats. (?) We are still not sure if this quercetin activates this or not.

This study presents significant findings regarding the neural toxicity induced by CdCl_2_ and the protective effects conferred by quercetin (QUR). The results demonstrate that CdCl_2_-induced hippocampal damage and associated memory deficits are linked to a marked impairment in hippocampal levels of peroxisome proliferator-activated receptor gamma (PPARγ), a key regulator of oxidative stress, inflammation, and apoptosis within neural tissues. Notably, activation of PPARγ by QUR fully mitigated the oxidative and inflammatory damage caused by Cd ions in the hippocampus, effectively restoring short-term and recognition memory functions. These effects were comparable to those observed with Pioglitazone, a selective PPARγ agonist. Importantly, this study provides new insight into the upstream mechanisms underlying the neuroprotective actions of QUR. These data also suggest that QUR’s antioxidant, anti-inflammatory, and anti-apoptotic effects in the CdCl_2_-treated brain are primarily mediated through the upregulation and nuclear activation of PPARγ. This conclusion is further substantiated by the observation that inhibition of PPARγ using GW9662, a selective antagonist, abolished the neuroprotective effects of QUR and reversed its beneficial impact on hippocampal morphology and memory (Figure 5).

Oxidative stress and inflammation are well-established contributors to neurodegeneration in numerous neurological disorders. The detrimental role of these factors in synaptic plasticity, long-term potentiation (LTP), memory impairment, and neurodegeneration has been extensively documented [5,6]. In this context, Cd ions have been shown to disrupt mitochondrial electron transport, replace essential zinc ions, deplete antioxidants, and increase reactive oxygen species (ROS) production, thereby impeding neural energy metabolism and calcium signaling [28,29]. Furthermore, CdCl_2_ exposure leads to diminished nuclear activation of Nrf2 and enhances the activation of NF-κB, both of which exacerbate oxidative stress and inflammation. The resulting accumulation of ROS and inflammatory cytokines contributes to intrinsic cell apoptosis by promoting DNA damage, increasing p53 expression, and upregulating Bax, which translocates to the nucleus and induces the release of cytochrome c, triggering apoptotic cascades [30]. Moreover, Cd ions disrupt synaptic plasticity and memory function by reducing brain-derived neurotrophic factor (BDNF) levels and impairing cholinergic function, either by promoting acetylcholine (Ach) degradation through acetylcholinesterase (AchE) upregulation or by inhibiting Ach synthesis via suppression of choline acetyltransferase (ChAT) [17,31,32].

In alignment with previous studies, this study established an animal model of hippocampal damage induced by chronic CdCl_2_ administration over 30 days. The observed deficits in memory, evidenced by reductions in BDNF, ACh, AChE, and ChAT levels, along with altered performance in cognitive assessments such as the Morris Water Maze (MWM), Novel Object Recognition Test (NORT), and Passive Avoidance Learning Test (PALT), substantiate previous research employing similar methodologies [17,27,33,34]. Furthermore, the elevated concentrations of malondialdehyde (MDA), 8-hydroxy-2′-deoxyguanosine (8-OHdG), TNF-α, IL-6, Bax, and caspase-3, in conjunction with diminished levels of Bcl-2, glutathione (GSH), superoxide dismutase (SOD), and heme oxygenase-1 (HO-1) in the hippocampi of CdCl_2_-treated rats, strongly implicate the activation of oxidative stress, inflammatory, and apoptotic pathways in the neurotoxic effects of CdCl_2_. Moreover, these alterations were linked to suppressed Nrf2 activation and enhanced NF-κB activation, corroborating earlier studies that described similar molecular changes in response to Cd exposure [3,35,36,37,38,39,40,41].

In contrast, this study also reveals the potential of QUR to attenuate CdCl_2_-induced hippocampal damage and prevent memory decline in rats. QUR achieved this by mitigating oxidative stress, inflammation, and apoptosis, particularly by suppressing lipid peroxidation, activating Nrf2, inhibiting NF-κB, upregulating BDNF, and restoring normal cholinergic function through modulation of Ach and its associated enzymes. The neuroprotective effects observed align with prior studies demonstrating that quercetin (QUR) mitigates hippocampal and cortical degeneration in a range of experimental models of neurodegenerative disorders, including aging, Alzheimer’s disease (AD), stroke, Parkinson’s disease (PD), as well as in cases of intoxication with heavy metals and other neurotoxic agents [11,12,13,14,15,16,17]. The neuroprotective mechanisms of QUR are multifaceted and not entirely understood, but they are largely attributed to the attenuation of oxidative stress and inflammation. Notably, QUR’s antioxidant activity is facilitated by its unique chemical structure, which contains multiple phenolic hydroxyl groups and a conjugated double bond, enabling it to scavenge ROS efficiently [9]. Additionally, its anti-inflammatory effects are likely mediated through the suppression of immune cell infiltration, inflammatory cytokine production, and activation of key signaling pathways, including NF-κB, NLRP3 inflammasomes, and TLR2/MyD88/NF-κB signaling, while simultaneously activating SIRT1, Nrf2, and AMPK [11,12,13,14,15,16,17,42,43,44,45,46,47,48]. Furthermore, QUR acts as a natural inhibitor of AchE, enhancing cell survival, synaptic plasticity, and memory function through modulation of acetylcholine levels and signaling pathways, including the BDNF, PI3K/Akt, and MAPK/ERK pathways [49,50,51,52].

PPARγ agonists, including thiazolidinedione derivatives like Pioglitazone, are widely used in clinical practice. PPARγ is abundantly expressed in various brain regions, including the hippocampus, thalamus, and basal ganglia, and is present in neurons, microglia, and astrocytes [7,42]. Beyond its role in metabolic regulation, PPARγ serves as a neuroprotective agent capable of attenuating cognitive deficits and neural damage in a variety of animal models of neurodegenerative diseases [10,42,43,44]. A novel finding of this study is the significant reduction in the expression, translation, and nuclear activity of PPARγ in the hippocampi of CdCl_2_-treated rats. The findings of this investigation also demonstrated that activation of PPARγ with Pioglitazone not only attenuated hippocampal damage by reducing oxidative stress and inflammation but also improved spatial and recognition memory. This effect was accompanied by restored levels of BDNF and Ach, as well as enhanced synaptic plasticity and mitochondrial function through increased Nrf2 activation and reduced NF-κB levels. These findings suggest that PPARγ activation exerts antioxidant, anti-inflammatory, and anti-apoptotic effects in the brain by modulating key transcription factors involved in these processes.

This study is the first to demonstrate that CdCl_2_-induced toxicity is associated with downregulating the transcription and nuclear activity of PPARγ’s in the hippocampus, consistent with previous reports showing depletion of PPARγ in the cortices and hippocampi of various animal models of neurodegeneration, including ischemia, AD, PD, diabetes mellitus, and ALS [7,53,54,55,56,57]. Furthermore, treatment with Rosiglitazone has been shown to improve memory function, synaptic plasticity, and long-term potentiation (LTP) in aged rats and prevent hippocampal damage in AD models [55,56,57,58,59,60]. Similarly, Pioglitazone has been demonstrated to attenuate cognitive deficits and reduce oxidative stress and inflammation in AD, dementia, and diabetes mellitus models, as well as in models of LPS-induced neuroinflammation [28,54,55,58,59]. Clinical studies have confirmed the efficacy of Pioglitazone in improving memory and cognitive function in patients with AD and multiple sclerosis [60,61,62].

Additionally, PPARγ neuroprotection is linked to its regulation of glucose receptors, upregulation of BDNF, enhancement of mitochondrial biogenesis, and activation of antioxidant and anti-apoptotic pathways such as Nrf2 and Wnt/β-catenin [7,63,64,65,66,67,68]. Building upon prior work and others’ findings, this study further elucidates the role of PPARγ in mediating QUR’s neuroprotective effects in CdCl2-intoxicated rats. The ameliorative effects of QUR on hippocampal damage, inflammatory markers, Nrf2/antioxidant axis, BDNF levels, apoptosis, and cholinergic markers were found to be predominantly mediated by PPARγ activation. These findings were corroborated by the blockade of PPARγ activation using GW9662, which eliminated the neuroprotective benefits of QUR.

However, the mechanism by which QUR activates PPARγ remains unexplored and may require further investigation. Previously, our team has shown that treatment with QUR attenuates CdCl_2_-mediated hippocampal damage in rats by activating SIRT1 [17]. The relationship between SIRT1 and PPARγ is complex and somewhat paradoxical. On one hand, SIRT1 can directly inhibit the transcription and activation of PPARγ [69]. On the other hand, SIRT1 can deacetylate PGC1α, which subsequently enhances the transcriptional activity of PPARγ [69]. Moreover, PPARγ itself can bind directly to the SIRT1 promoter and inhibit its transcription [70]. Concurrently, AMPK serves as a potent activator of SIRT1 [71,72]. In both clinical and experimental contexts, PPARγ agonists like Troglitazone and Pioglitazone have been shown to phosphorylate and activate AMPK independently of PPARγ activity modulation [72]. These agonists can improve insulin signaling, regulate glucose and lipid metabolism, and offer protection against Type 2 diabetes mellitus (T2DM) by stimulating AMPK activity in adipose tissue, liver, and muscle, irrespective of PPARγ activation [72,73,74]. Given these observations, it remains unclear whether SIRT1 or PPARγ is the primary upstream target of QUR. It is plausible that QUR activates SIRT1 in the hippocampus through the PPARγ/AMPK axis, or alternatively, QUR may stimulate PPARγ via the SIRT1/PGC1α pathway. Consequently, further studies are essential to clarify these interactions and to determine the primary neural regulator through which QUR exerts its effects.

## 4. Materials and Methods

### 4.1. Animals

Adult male Wistar rats (220 ± 20 g, 7–8 weeks of age) were collected from the animal center at King Saud University, KSA. The animals were housed in a separate room at 22 ± 2 °C. The light was always controlled on a 12 h-cycle. Throughout the experiments, all rats were supplied with a sterile standard diet and tap water with 24-h free access. Drug administration, anesthesia, euthanasia, and blood and tissue collections were performed following the international principles of laboratory animal care published by NIH (number 82-23, 1985) after being approved by the institutional ethical committee.

### 4.2. Experimental Design

In this study, a cohort of 60 male rats was randomly allocated into five distinct experimental groups, each consisting of 12 animals. The groups were categorized as follows: (1) Control Group: This group served as the baseline and received a daily oral administration of the vehicle (0.5% carboxymethylcellulose) without any active treatment. (2) CdCl_2_-Treated Group: Rats in this group were subjected to chronic oral administration of cadmium chloride (CdCl_2_) at a dose of 0.5 mg/kg per day to induce neurotoxic effects. (3) CdCl_2_ + Pioglitazone Group: In addition to receiving CdCl_2_ (0.5 mg/kg), this group was administered Pioglitazone, a selective peroxisome proliferator-activated receptor gamma (PPAR-γ) agonist, at a dose of 10 mg/kg per day, to assess its potential neuroprotective properties. (4) CdCl_2_ + Quercetin (QUR)-Treated Group: This group received both CdCl_2_ (0.5 mg/kg) and quercetin (QUR) at a dose of 25 mg/kg per day to evaluate the protective effects of QUR against CdCl_2_-induced neurotoxicity. (5) CdCl_2_ + QUR + GW9662 Group: Rats in this group were treated with CdCl_2_ (0.5 mg/kg) and QUR (25 mg/kg), and also received GW9662, a PPAR-γ antagonist, administered intraperitoneally at a dose of 1 mg/kg per day, to determine the role of PPAR-γ inhibition in modulating the effects of QUR.

For all oral treatments (QUR, Pioglitazone, and CdCl_2_), an intragastric tube was used for precise dosing. GW9662 was given 1 h before treatment with QUR, which was also given 2 h before treatment with Cd. All treatments, including QUR, Pioglitazone, and GW9662, were administered for 30 days on a daily basis according to the above sequence. The treatments were administered for a predetermined duration, with all groups being monitored for physiological and behavioral changes.

### 4.3. Dose Selection and Protocol Regimen

As per the results from our labs and the results of other reports, treatment with CdCl_2_ solution (0.5 mg/kg) for 30 consecutive days impairs memory and learning function and induces hippocampal degeneration in rats [17,27,33]. Oral treatment with QUR at a dose of 25 mg/kg was fully safe in control rodents and attenuated the functional and structural neurological changes in these CdCl_2_-treated rats [17]. The administration of Pioglitazone (10 mg/kg) via repeated oral dosing was employed to enhance the activation of PPARγ across the brain and other tissues. This regimen has demonstrated protective effects on renal, hepatic, ocular, and neurological systems in various experimental models [75,76,77,78,79,80]. The repeated in vivo intraperitoneal administration of GW9662 at 1 mg/kg has been used in several studies to completely inhibit PPARγ in the brains, kidneys, and retinas of rodents [76,77,79,81]. We have also confirmed this in preliminary data where the activity of PPARγ was almost inhibited by 90% in the brain tissue using this protocol of administration (i.e., at least 2 h before treatment with QUR).

### 4.4. Assessment of Neurological Score

The assessment of the neurological score was performed as described by others [82]. This test assesses the ability of each rat to perform 10 tasks that reflect its awareness, alertness, balance, motor function, and general behavior. A score of 0 was given if the rat performed the task, and a score of 1 was given if it failed to perform the task. Therefore, the maximum score was 10, and a higher score indicates a worse neurological assessment. These tasks are well described in [82] and include exiting a circle 30 cm in diameter, mono-/hemiparesis of the legs, walking in a straight line, exhibiting a startled reflex to a loud hand clap, showing seeking behavior in the environment, balancing on a beam 7 mm in width for 10 s, balancing a round stick for 10 s, and 3 other independent tasks involving crossing 3 different beams (30 cm × 3 mm, 30 cm × 2 cm, and 30 cm × 1 cm). The test was conducted over the last three days of the experiment, and average readings were taken for each rat.

### 4.5. Assessment of Memory Function

Spatial and short-term memory in rats were evaluated using the Morris Water Maze (MWM) and the Passive Avoidance Learning Test (PALT), respectively, while the ability to recognize objects was assessed through the Novel Object Recognition Test (NORT). These tests are well described in our laboratories and have been used to test memory function by other researchers [82,83].

### 4.6. The Morris Water Maze (MWM)

The MWM was performed every morning in a circular swimming pool (1 m height × 60 cm depth), which was hypothetically divided into 4 quadrants and camouflaged with milk; a hidden rescue platform was embedded in one of the quadrants (1.8 cm below the water level). During the test, the rat is released from one quadrant and swims to find the hidden platform within 100 s. If the rat finds it, it is allowed to stay there for 15 s before returning it to its cage. If the rat does not find it, it is directed towards it. This process was repeated over 4 days (days 26–29), each comprising 3 trials. On day 30, a probe trial was performed in which the rats were given an opportunity to retake the test without the platform. The number of crossings over the platform’s original location was recorded for each rat.

### 4.7. Passive Learning Avoidance Test (PALT)

At midday on day 29, the PALT was conducted in a wooden box (50 × 50 × 35 cm) with an electrical mesh floor; the box was comprised of two rooms, one illuminated and the other dark, separated by a door. Initially, each rat underwent an exploration of 3 trials, each of 5 min (separated by 10 min). In the course of these trials, the rat was initially placed in the lit section with the door open and allowed to freely enter the dark compartment to explore. Following this, a fourth trial was conducted in which a similar procedure was used. However, in this trial, once the rat entered the dark area, the door was closed, and the rat was subjected to a 2-s electrical foot shock at 50 Hz. Afterward, the rat was returned to its cage, and the test was repeated after 3 h. Rats with functional short-term memory typically avoid entering the dark area. The apparatus was cleaned between each trial.

### 4.8. The Novel Object Recognition Test (NORT)

The NORT was also conducted on day 30 in a wooden box with an open roof (50 × 50 × 50 cm). The test comprises two trials; in the first trial, each rat was placed in the box and allowed to freely explore two identical objects (red plastic cubes) for 15 min. Two hours later, the same rat was reintroduced into the wooden box, where it was given the opportunity to explore two objects for a duration of 5 min—one familiar (a red plastic cup) and the other novel (a glass cube). The time each rat spent exploring the novel object was recorded. Rats with intact recognition memory spent more time exploring the new object in the second trial.

### 4.9. Collection of Brains and Hippocampi

All rats from the experimental groups were euthanized using ketamine (80 mg/kg body weight) and decapitated. The skulls were opened, and the brains were carefully extracted while kept on ice. The brains of the first four rats were promptly preserved in 10% buffered formalin and sent to the pathology lab for routine histological analysis. The hippocampi of the remaining eight rats were quickly harvested by a trained anatomist using a dissecting microscope (INFITEK INC., Jinan, Shandong, China) on ice, snap-frozen in liquid nitrogen, and stored at −80 °C for further analysis.

### 4.10. Enzyme-Linked Immunosorbent Assays (ELISA)

Parts of the collected hippocampi (40 mg) of each rat were homogenized using a Q-Sonica homogenizer (Newtown, CT, USA) for 20 s in ice at 30 Hz in a neural cell lysis buffer (cat # 87792, Thermo Scientific, Waltham, MA, USA). Other fractions of the hippocampi were used for the extraction of the nuclear and cytoplasmic proteins (NE-Per) (cat # 78833, Thermo Scientific, Waltham, MA, USA). All samples were centrifuged for 15 min at 11,600× *g* to collect the supernatants. Protein concentrations in the samples were determined using a Pierce BCA assay kit (Cat # 23225, Thermo Scientific, Waltham, MA, USA). The supernatants of the total hippocampal tissue extract were subjected to ELISA to measure the levels of tumor necrosis factor-alpha (TNF-α), malondialdehyde (MDA), total glutathione (GSH), acetylcholine (Ach), acetylcholine esterase (AchE), acetylcholine acetyltransferase (AchT) superoxide dismutase (SOD), Heme oxygenase-1 (HO-1), brain-derived neurotrophic factor (BDNF), Bax, Bcl2, and caspapse-3. The ELISA also measured the levels of Nrf2, PPARα, and NF-κB in the nuclear extracts. All kits were specific to rats and were purchased from MyBioSource (San Diego, CA, USA). All samples were analyzed in duplicate for n = 8 rats/group using an ELx800 microplate reader (BioTek Instruments Inc., Winooski, VT, USA).

### 4.11. Real-Time PCR (qPCR)

For the amplification of PPARγ (NM_013124.3), the primers used were (F: 5′-CATGACCAGGGAGTTCCTCAA-3′; R: 5′-AGCAAACTCAAACTTAGGCTCCAT-3′, producing a 73 bp fragment), and for GAPDH (NM_017008.3), the primers were (F: 5′-CCATCAACGACCCCTTCATT-3′; R: 5′-CACGACATACTCAGCACCAGC-3′, producing a 193 kD product). Briefly, total RNA was extracted from 30 mg of frozen hippocampal tissue using the TRIZOL reagent. The first cDNA strand was synthesized using a commercially available kit (Catalog # ab 286905). Primers for Nrf2, NF-κB, and β-actin (the reference gene) were sourced from ThermoFisher. Real-time PCR amplification was performed using a CFX96 real-time PCR machine (BioRad, Hercules, CA, USA) with the Ssofast Evergreen Supermix kit, following the manufacturer’s guidelines. Expression levels of Nrf2 and NF-κB were normalized to β-actin mRNA levels, and data were analyzed using the 2^ΔΔCT^ method. Six samples were analyzed per group.

### 4.12. Determination of PPARα Nuclear Activity

The binding activity of PPARγ with DNA was measured as previously described by others, using a rat-specific non-radioactive colorimetric transcription activity kit (Cat. # 10006855 Cayman, Ann Arbor, MI, USA). The principle of the test is based on the primary antibody detection of PPARγ in the nuclear extract that binds to the specific double-stranded DNA that is immobilized in the wells. The detection is achieved by adding an HRP-conjugated secondary antibody and reading the signal at 450 nm. The protocol was conducted for 8 samples for each group, following the manufacturer’s instructions.

### 4.13. Histological Assessment

The rat brains were immersed in 10% buffered formalin for 20 h to fix them, followed by deparaffinization using pure xylene. Tissue rehydration was performed by immersing the brain tissues in descending alcohol concentrations (100–70%). Small sections of 4–5 μm were obtained using the rotatory microtome; these sections were then placed on glass slides and routinely stained with Harris hematoxylin and Eosin. Mounting media and coverslips were added, and all slides were examined and photographed using a light microscope (Leica DM6, Leia microsystem, Wetzlar, Germany).

### 4.14. Statistical Analysis

The data for all parameters were gathered and processed using GraphPad Prism software (version 8, GraphPad Software Inc., La Jolla, CA, USA). Statistical significance was assessed via one-way ANOVA, with Tukey’s test applied as a post hoc analysis (*p* < 0.05). All results are presented as the mean ± standard deviation (SD).

## 5. Conclusions

In summary, this study provides compelling evidence that CdCl2-induced hippocampal damage and cognitive impairments are closely associated with the downregulation of PPARγ, a critical regulator of oxidative stress, inflammation, and apoptosis in the brain. Activation of PPARγ by quercetin (QUR) effectively attenuates these neurotoxic effects, restoring normal hippocampal function and memory performance. The neuroprotective effects of QUR were shown to be primarily mediated through the upregulation and nuclear activation of PPARγ, highlighting the pivotal role of this receptor in mitigating oxidative damage and inflammation in the brain. Furthermore, these findings suggest that QUR’s ability to activate PPARγ represents a novel and crucial upstream mechanism underlying its neuroprotective actions. These results not only contribute to our understanding of the molecular mechanisms driving CdCl_2_-induced neurotoxicity but also suggest that PPARγ modulation may represent a promising therapeutic target for mitigating neurodegenerative processes associated with heavy metal toxicity. Given the complexity of the interactions between PPARγ, SIRT1, and AMPK, further investigations are necessary to elucidate the precise pathways through which QUR activates PPARγ and to determine its potential therapeutic applications in neurodegenerative diseases.

### Study Limitations

While this study provides valuable insights into the neuroprotective role of PPARγ activation by quercetin (QUR) in alleviating CdCl_2_-induced hippocampal damage, several limitations should be considered. First, this study was conducted in an animal model, and further research is needed to validate the findings in human subjects or more complex in vitro models, which could provide a better understanding of the translational potential of these results. Second, while we demonstrated that PPARγ activation mediates the neuroprotective effects of QUR, the exact molecular interactions between PPARγ, SIRT1, and AMPK, as well as their contribution to the observed effects, remain to be fully elucidated. Future studies should focus on identifying the specific signaling pathways involved in QUR’s modulation of PPARγ, SIRT1, and AMPK and whether these pathways could be targeted for therapeutic interventions. Additionally, long-term studies examining the chronic effects of QUR on neurodegeneration and cognitive function are needed to determine its sustained efficacy and potential therapeutic relevance in treating neurodegenerative diseases. Lastly, research exploring the potential synergistic effects of QUR in combination with other PPARγ modulators or neuroprotective agents may open new avenues for developing more effective treatment strategies for metal-induced neurotoxicity and other neurodegenerative conditions.

## Figures and Tables

**Table 1:** Hippocampal oxidative stress and inflammation profiles in different rat groups.

Parameter	Control	CdCl_2_	CdCl_2_ + QUR	CdCl_2_ + QUR + GW9662	CdCl_2_ + Pioglitazone
Total Levels					
MDA (nmol/g protein)	0.65 ± 0.08	2.88 ± 0.39 ***	0.89 ± 0.06 *^,###^	2.62 ± 0.27 ***^,&&&^	0.71 ± 0.09 ^###,$$$^
GSH (µg/g protein)	76.5 ± 6.8	27.8 ± 2.4 ***	79.4 ± 7.1 ^###^	31.4 ± 3.3 ***^,&&&^	71.4 ± 8.3 ^###,$$$^
SOD (U/g protein)	29.7 ± 2.9	11.3 ± 1.6 ***	26.7 ± 2.1 ^###^	13.5 ± 1.8 ***^,&&&^	31.2 ± 3.7 ^###,$$$^
HO-1 (U/g protein)	24.5 ± 2.7	8.7 ± 0.92 ***	22.4 ± 3.2 ^###^	9.4 ± 0.82 ***^,&&&^	26.5 ± 2.1 ^###,$$$^
8-OHdG (pg/g protein)	320.2 ± 27.7	738.2 ± 66.4 ***	305 ± 37.3 ^###^	822.5 ± 73.4 ***^,&&&^	352 ± 44.7 ^###,$$$^
RAGE (ng/g protein)	7.6 ± 0.64	25.7 ± 3.1 ***	9.4 ± 0.83 *^,###^	27.5 ± 2.6 ***^,&&&^	8.5 ± 0.67 ^###,$$$^
TNF-α (pg/g protein)	10.6 ± 0.97	46.5 ± 5.2 ***	17.5 ± 1.8 **^,###^	51.2 ± 6.4 ***^,&&&^	16.8 ± 1.4 ^###,$$$^
IL-6 (pg/g protein)	2.4 ± 2.4	37.6 ± 3.1 ***	6.7 ± 0.72 ***^,###^	41.2 ± 5.7 ***^,&&&^	8.5 ± 0.91 ^###,$$$^
Nuclear levels					
Nrf2 (pg/g protein)	325.5 ± 24.5	122.5 ± 11.9 ***	211.3 ± 22.8 ^###^	143.6 ± 15.3 ***^,&&&^	318.7 ± 28.4 ^###,$$$^
NF-κβ p65 (pg/g protein)	219.2 ± 18.9	513.1 ± 48.4 ***	245.8 ± 22.1 *^,###^	489.7 ± 43.2 ***^,&&&^	239.9 ± 26.6 ^###,$$$^

Data are presented as the means ± SD (n = 8/group). The values were significantly different at *p* < 0.05. *^,^ **^,^ ***: Compared to control (*p* < 0.05, 0.01, & 0.001 Statistical significance was determined at *p* < 0.05. ***: Compared to the control group (*p* < 0.001); ^###^: Compared to CdCl_2_-treated rats (*p* < 0.001); ^&&&^: Compared to CdCl_2_ + QUR-treated rats (*p* < 0.001); and ^$$$^: Compared to CdCl_2_ + QUR + GW9662 (*p* < 0.001). Tumor necrosis factor-alpha (TNF-α), malondialdehyde (MDA), total glutathione (GSH), superoxide dismutase (SOD), Hemeoxygenase-1 (HO-1), receptors of advanced glycation end products (RAGE), and 8-hydroxy-2′-deoxyguanosine (8-OHdG).

**Table 2:** The neurological scores and levels of BDNF and cholinergic neurological markers in the hippocampi of all groups of rats.

Parameter	Control	CdCl_2_	CdCl_2_ + QUR	CdCl_2_ + QUR + GW9662	CdCl_2_ + Pioglitazone
BDNF (ng/g protein)	104.2 ± 12.4	43.2 ± 4.6 ***	87.9 ± 6.8 **^,###^	38.5 ± 3.1 ***^,&&^	91.9 ± 7.7 ^###,$$$^
Ach (μmol/g protein)	4.56 ± 0.27	1.63 ± 0.26 ***	3.92 ± 0.41 *^,###^	1.29 ± 0.22 ***^,#,&&&^	4.34 ± 0.62 ^###,$$$^
AchE (pg/g protein)	18.5 ± 1.7	46.5 ± 6.4 ***	22.5 ± 1.9 *^,###^	51.8 ± 5.8 ***^,#,&&&^	19.3 ± 1.3 ^###,$$$^
ChAT (mU/gprotein)	11.42 ± 1.3	4.52 ± 0.44 ***	10.21 ± 1.37 ^###^	3.42 ± 0.45 ***^,#,&&&^	9.88 ± 0.86 ^###,$$$^

Data are presented as the means ± SD (n = 8/group). The values were significantly different at *p* < 0.05. *^,^ **^,^ ***: Compared to control (*p* < 0.05, 0.01, & 0.001 Statistical significance was determined at *p* < 0.05. ***: Compared to the control group (*p* < 0.001); ^#, ###^: Compared to CdCl_2_-treated rats (*p* < 0.001); ^&&, &&&^: Compared to CdCl_2_ + QUR-treated rats (*p* < 0.001); and ^$$$^: Compared to CdCl_2_ + QUR + GW9662 (*p* < 0.001). Brain-derived neurotrophic factor (BDNF), acetylcholine (Ach), acetylcholine esterase (AchE), and acetylcholine acetyltransferase (ChAT).

## Data Availability

Data is contained within the article.

## Abbreviations

The following abbreviations are used in this manuscript:

PPARγ	Peroxisome proliferator-activated receptor gamma
QUR	Quercetin
CdCl_2_	Cadmium chloride
